# A panoramic spectrum of complex interplay between the immune system and IL-32 during pathogenesis of various systemic infections and inflammation

**DOI:** 10.1186/s40001-015-0083-y

**Published:** 2015-01-28

**Authors:** Babar Khawar, Muddasir Hassan Abbasi, Nadeem Sheikh

**Affiliations:** Cell and Molecular Biology Lab, Department of Zoology, University of the Punjab, Quaid-e-Azam Campus, Lahore, Pakistan; Department of Zoology, Governments. College of Science, Wahdat Road, Lahore, Pakistan

**Keywords:** Auto immune disease, Inflammatory bowel disease, Interleukin-32, Rheumatoid arthritis

## Abstract

Cytokines have always been of great interest due to their vast potential and participation in the progression and pathogenesis of various ailments. Interleukin-32 (IL-32) is a recently identified cytokine, whose gene is located on human chromosome 16 p13.3, with eight exons and six splice variants (IL-32α to IL-32ζ). IL-32α, the most abundant form, is secreted by different types of cells including T cells, natural killer (NK) cells, monocytes, endothelial cells and epithelial cells. It acts as a preferential mediator and effector of abnormal immune responses to multiple inflammatory and auto immune diseases including rheumatoid arthritis, chronic obstructive pulmonary disease (COPD), inflammatory bowel disease (IBD), etc. It was found to stimulate the induction of various chemokines, pro-inflammatory cytokines including IL-1β, IL-6, IL-8, TNF-α and macrophage inflammatory protein-2 (MIP-2). Hence, IL-32 mediates the crucial interplay among immune system and body cells during pathogenesis of various insults. The aim of the present effort is to summarize the role, mechanism of pathogenesis and potential therapeutic applications of IL-32 in different systemic infections and diseased conditions.

## Introduction

Cytokines are small, pleiotropic, nonstructural soluble factors (probably polypeptides/proteins) with molecular weights ranging between 8 to 40,000 Da. Every cell is capable of producing cytokines and can respond to them. Cytokines are principally involved in homeostatic mechanisms by mediating and regulating inflammatory/ immune responses to various insults like diseased conditions or infections and affect cellular interactions and cell communication system. These peptides could be autocrine, paracrine and perhaps even endocrine regarding their site of action [1,2]. These are principally classified into various classes on the basis of their biological roles. The term cytokines includes lymphokines, monokines, chemokines and interleukins made and secreted by a variety of immune system components, specifically, lymphocytes, monocytes and leukocytes. The aim of the current review is to emphasize the existing therapeutic potential and future perspective of interleukin-32 (IL-32). The extraordinary qualities of this interleukin have prompted their application in the field of medical biology.

**Figure 1:**
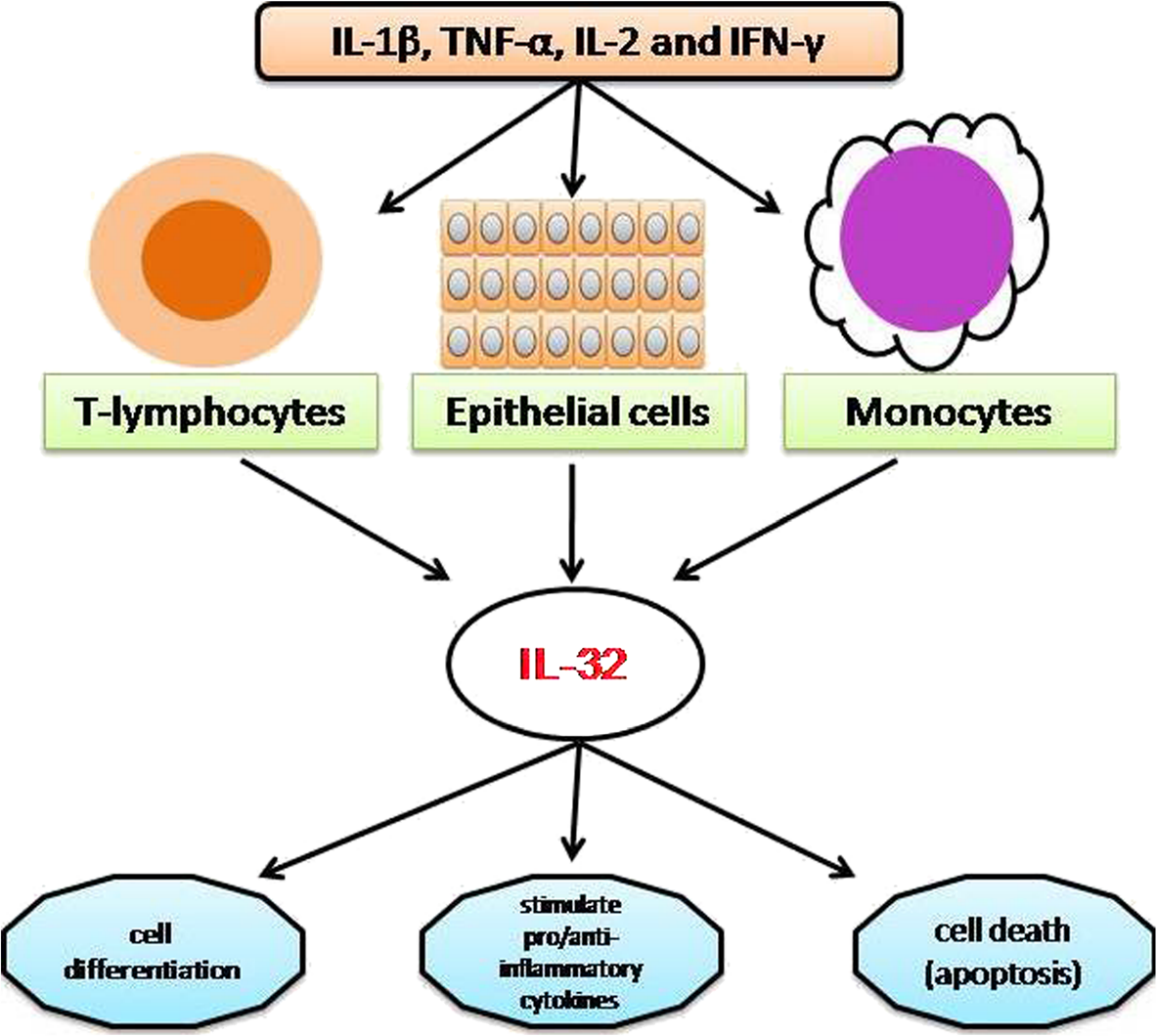
**Production of IL-32 by various body cells, namely, epithelial cell lines, T-lymphocytes and monocytes, and its involvement in different cellular processes including cell differentiation and cell death, as well as interactions with the immune system.**

Interleukin-32 (IL-32), a recently described cytokine (previously called natural killer cell transcript 4 (NK4)), found originally as a transcript that is in a cDNA library derived from IL-2 activated natural killer cells [3], as well as being expressed selectively in T-lymphocytes, monocytes and epithelial cell lines [4-6]. It is an important player in innate and adaptive immune responses *in vitro* [4], and its production is predominantly induced by IL-1β, TNF-α, IL-2 or IFN-γ in blood monocytes and epithelial cells [3,5,7]. IL-32 is a pleiotropic cytokine that is involved in number of biological functions including cell differentiation [8-10], stimulation of pro- or anti-inflammatory cytokines [11-13] and cell death, especially apoptosis (Figure 1) [14,15].

## Review

Human recombinant IL-32 does not exhibit similarities with known cytokine families, yet several properties are typical of a pro-inflammatory cytokine [5,16,17]. It was discovered accidently while studying the genes induced by IL-18 and was found to stimulate the production of various chemokines, pro-inflammatory cytokines including IL-1β, IL-6, IL-8, TNF-α and macrophage inflammatory protein-2 (MIP-2) [5,10,17,18].

Inflammation or infection with various pathogens including *Mycobacterium tuberculosis*, Epstein-Barr virus (EBV), human immunodeficiency virus (HIV) and influenza A virus have been reported to induce the expression of IL-32 [19-22]. The IL-32 gene is located on human chromosome 16 p13.3, which is organized into eight exons with six splice variants of the gene; these variants have been described as IL-32α, IL-32β, IL-32γ, IL-32δ, IL-32ε and IL-32ζ [23,24], of which, IL-32α is the most abundant transcript [25].

Anti-tumor activity of NK cells is provoked by IL-12 and IL-18, both of which induce IL-32 production that stimulates TNF-α synthesis enhancing NK apoptotic activity [3,5,6,26]. IL-32 was found in cytosol as well as in the nucleus. Park *et al.* [27] reported that IL-32 enhances the anti-tumor activity specifically for NK-92 cells upon introduction of the death receptor and the activation of caspase-3 pathway in cancer cells.

IL-32 has been reported to play a key role in the pathogenesis of various disorders, including infectious autoimmune and inflammatory diseases.

### IL-32 in rheumatoid arthritis

Rheumatoid arthritis (RA) is a chronic autoimmune disease that is often associated with inflammation and joint destruction, which ultimately results in significant disability. Cagnard *et al.* [28] reported an elevation in IL-32 expression in patients with rheumatoid arthritis (RA) in contrast to osteoarthritis [28], and the severity of the symptoms was found to be correlated with high degree of expression of TNF-α, a potent inducer of IL-32 mRNA expression in human synovial fibroblasts. Overexpression of IL-32 in turn stabilized the mRNA transcripts of other cytokines, namely, those for TNF-α, IL-1β and IL-8.

**Figure 2:**
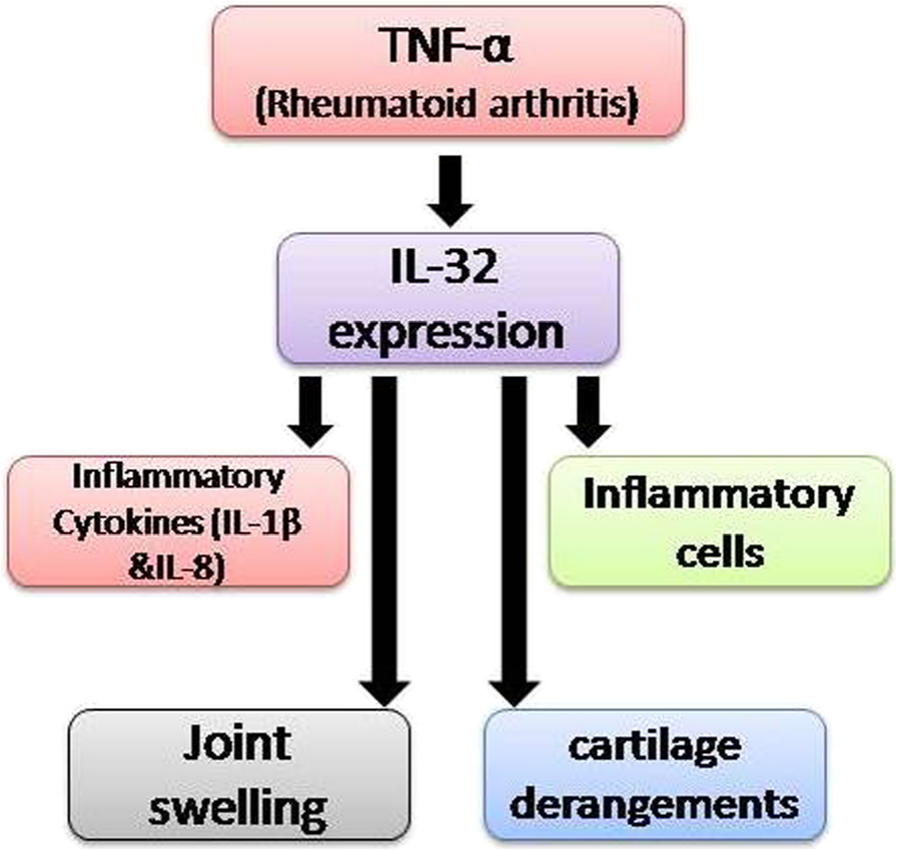
**Mechanism of pathogenesis of autoimmune disease, rheumatoid arthritis (RA).** In a classical pathway, pro-inflammatory cytokines TNF-α induce expression of IL-32, which leads not only to progression of disease but may cause injury.

In another study on anti-TNF-α treatment in patients with RA, synovial knee biopsies showed a significant decrease in IL-32 expression [11]. Similarly, human IL-32 leads to joint swelling and recruitment of inflammatory cells, along with cartilage derangements, when injected in joints of naïve mice. In contrast, in a TNF-α deficient mice model, joint swelling and influx of inflammatory cells have been drastically decreased (Figure 2) [22].

In a study to explore the role of IL-32 in RA, CD14^+^ monocytes and synovial tissue were analyzed from healthy volunteers and RA patients. The IL-32γ level was found significantly upregulated in RA patients in accordance with results of an experimental model of inflammatory arthritis in mice in which the administration of IL-32 aggravated disease conditions [8,29,30]. Synergism between the soluble receptor activator of nuclear factor κ-B ligand (sRANKL) and IL-32γ was demonstrated. In IL-32γ treated cultures, the presence of sRANKL aggravates the activity of osteoclasts, as well as increases the resorption of tissues, compared to IL-17 [8]. Presence of significantly high levels of IL-32 in synovial tissue biopsies of RA as compared to its absence in osteoarthritic (OA) patients confirmed that IL-32 is potent mediator of the active osteoclastogenic activity [22]. This extraordinary interleukin was also found to be involved in the release of prostaglandin E2 from human blood monocytes and mouse macrophages. IL-32 mediated cell influx and joint swelling was found to be reduced in TNFα-deficient mice, which suggest that the expression of this cytokine is TNFα-dependent in RA [22]. The signal pathway of TNFα-dependent mediation of IL-32 expression in RA was explored by Moon *et al.* They reported the involvement of spleen tyrosine kinase (Syk)/ protein kinase Cδ (PKCδ)/c-Jun N-terminal kinase (JNK) pathways in the regulation of IL-32 induction by TNF-α in synovial fibroblasts [29]. An upregulated level of IL-32 was found in cases of RA fibroblast-like synoviocytes (FLS), while it was absent in biopsies of OA. This increased level of IL-32 was found to be suppressed by small interfering RNA (siRNA) of these enzymes as well as by the inhibitors of PKCδ, Syk and JNK. Recent research has revealed the key role of FLS in osteoclastic activity, as well as their role in pannus formation in joints [31]. Various factors and interactions such as cell-to-cell contacts, pathogen-associated molecular patterns (PAMPs), pattern-recognition receptors (PRRs), and cytokine environment, along with damage-associated molecular patterns (DAMPs), may be involved in the activation of FLS. Lipopolysaccharide (LPS), peptidoglycan and some other bacterial products have also been reported, which result in the activation of FLS activity by interacting with PRRs of these cells [32,33]. FLSs, in response to inflammatory stimuli, expressed a large number of PRRs, especially TLR2, TLR3 and TLR4, [34,35]. FLS-induced inflammatory response is mediated by synthesizing a variety of cytokines, prostanoids, chemokines, and nitric oxide (NO) [31]. TNF-α, IFN-γ, and PAMPS have been reported to regulate the secretion of IL-6, IL-8, and B-cell-activating factor by FLS [36,37]. IL-32 and IL-17 are two major inflammatory cytokines, which together are thought to play a similar role and synergistically involved in differentiation of osteoclasts. A few new genes that is, CXC chemokine receptor 4 (CXCR4), IL-32 and lamina propria lymphocytes (LPL) were confirmed due to expression of IL-17A and IL-17 F in combination with TNF-α in RA synoviocytes [38]. CD4^+^ T cells or dendritic cells and RA FLSs have been previously employed in various studies to reveal a reciprocal interaction between IL-32 and TNF-α depicting a TNF-α/IL-32/TNF-α-positive auto-inflammatory loop [11]. Anti-TNF-α treatment of RA patients has resulted in a significant reduction of IL-32 peptides in synovial tissue [11]. Recently, IL-32 g was reported to be involved in maturation and activation of immature dendritic cells (DCs), along with an increased Th1 and Th17 response by IL-12 and IL-6 [39]. Both IL-17 and IL-32 influence pathogenesis by TNF-R1 dependent/independent pathway by employing p300 and death-associated protein kinase 1 (DAPK-1) [40]. IL-32 and IL-17 can augment osteoclastogenesis by RANKL-dependent manner, as well as reciprocally affect each other's production in RA synovium.

IL-32β, δ, and γ mRNA overexpression in RA FLS is primarily induced by TNF-α, IFN-γ and toll-like receptor (TLR)-2, −3, and −4 ligands [41]. Mature IL-32 is expressed by various cells of the body due to intracellular polyriboinosinic polyribocytidylic acid (poly I:C) and TNF-α.

The overexpression of IL-32 in a number of diseases including asthma, inflammatory bowel disease (IBD) and RA has been reported to be induced by IL-18 [5].

### IL-32 in inflammatory bowel disease

**Figure 3:**
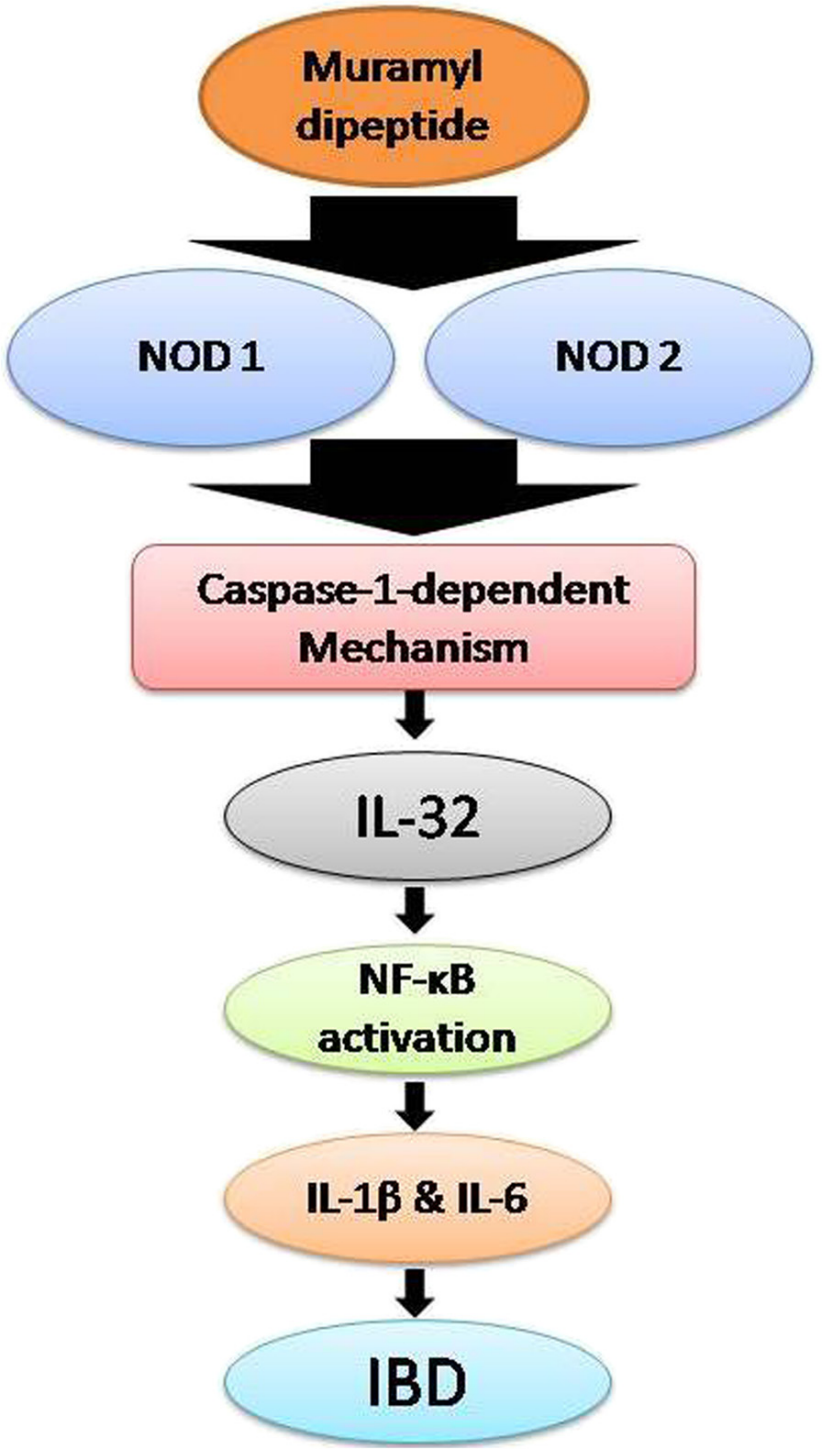
**Mechanism of pathogenesis of inflammatory bowel disease (IBD).** IBD pathogenesis is initiated by bacterial peptidoglycan, which induces caspase-1-dependent mechanism by NOD1 and NOD2, which enhanced the expression of IL-32. IL-32 initiates the NF-κB activation and a spectrum of various other inflammatory cytokines, and this activation ultimately result in IBD.

Inflammatory bowel disease (IBD), a chronic and degenerating inflammatory disorder of the gastrointestinal tract (GIT), has two common forms: ulcerative colitis (UC) and Crohn's disease (CD). The pathogenesis of disease has been found to be associated with following common symptoms: mild fever, abdominal pain, chronic diarrhea, and ulceration of colon/ rectum often resulting in rectal bleeding. In the case of CD, stenosis, fistulation, and abscesses may be sometimes associated with former symptoms, while in case of UC an additional problem megacolon has been reported. Currently, the exact mechanism involved in IBD is mysterious currently but role of unidentified components of gut microflora has been an established view in an abnormal cascade of inflammatory responses during this disease [42-46]. Netea *et al.* [17] found that a bacterial peptidoglycan (muramyl dipeptide) induced the expression of IL-32 with the nucleotide-binding oligomerization domain-containing protein 1 (NOD1) and the nucleotide-binding oligomerization domain-containing protein 2 (NOD2) through a caspase-1-dependent mechanism that ultimately induces activation of the nuclear factor kappa-light-chain-enhancer of activated B cells (NF-κB) and augments the production of IL-1β and IL-6 [17,47]. Recently, Crohn’s disease (CD) patients have been found to have an enhanced IL-1β and NF-κB activity due to mutation in NOD2. All of the above findings confirmed a key role of IL-32 in the progression and pathophysiology of IBD, especially in CD [48,49]. IL-32 and TNF-α seems to play a role in the pathogenesis of IBD, as IL-32 is found to be overexpressed in an amplified manner along with TNF-α (Figure 3) [25].

A new isoform of IL-32 has been identified in human colonic subepithelial myofibroblasts (SEMFs); this isoform lacks exon 3 and 4 of the IL-32γ isoform (longest isoform) and was named as IL-32ε [50]. An enhanced level of the transcript of IL-32ε was found in inflamed mucosa of IBD patients. TNF-α, in a time- and dose-dependent manner, was found to be an active inducer of transcript of this new IL-32ε [50]. It has been reported previously in a study in HT-29 cells, that transfection of IL-32ε results in an overall decrease of IL-8 transcript mediated by TNFα, but the expression of the shortest isoform IL-32α, which lacks exon 3 and 7, showed no effect on the IL-8 transcript. *In vivo* experiments were inspected to find out the role of IL-32 in intestinal inflammation by using an IL-32γ transgenic mouse (IL-32γ-TG) that expressed human IL-32γ. Considerable amounts of TNF-α were reported in the sera and colonic tissue of IL-32γ-TG mice while the mice remain healthy. Because of this enhanced pro-inflammatory cytokine, IL-32γ-TG exhibited a slightly early and greater acute inflammation as compared to wild type mice upon the dextran sodium sulfate (DSS)-induced colitis. Nevertheless, there is a lesser amount of colonic inflammation and better survival rate compared with wild-type mice after day 6. The colonic level of inflammatory cytokine related to attenuated tissue damage was significantly decreased in IL-32γ-TG-treated with DSS and the constitutive level of IL-32γ in colonic tissue was also decreased [51]. So conclusions can be drawn on how IL-32γ enhances the innate inflammation, as well as how it protects intestinal integrity.

IL-32 not only stimulates the production of various inflammatory cytokines with monocytes but also causes monocytes to differentiate into macrophage or dendritic cells (DCs) [10]. IL-32 stimulates neutrophils directly to produces IL-6 and IL-8 [8,51,52]. These differentiated macrophages and DCs are potent source of some very crucial inflammatory cytokines, that is, TNF-α, IL-1β, and IL-6, which recruit T-cells in the inflamed area in cases of IBD and CD. Differentiated DCs help in the proliferation of these T-cells to protect the host against the invading pathogens. This increase in the numbers of various immune cells without proper immune suppressor molecules leads to infiltration of neutrophils in an inflamed area, which results in the release of a variety of neutrophil proteinases such as cathepsin G, elastase, and proteinase 3 (PR3). These enzymes of serine proteinase family are powerful mediators of mucosal tissue injury exacerbating inflammation in CD and IBD. Though expression of IL-32 is elevated in the epithelial cells of inflamed mucosa from IBD and CD patients the biological role of IL-32 *in vivo* and *in vitro* was inconsistent. Eight different IL-32 mRNA transcripts give rise to five IL-32 isoforms (Kim S, 2014 unpublished data). The divergence of *in vitro* and *in vivo* data could be because of the fact that each researcher has studied a different IL-32 isoform.

In one study, the level of mRNA transcript and IL-32α protein was found to be significantly high in the IBD patients with inflamed epithelial mucosa compared to normal individuals [48]. Further studies suggested that IL-32 interacts with various other cytokines including IL-1β, TNF-α and IFNγ and plays an important role in pathophysiology of IBD and CD [53-55]. There is a need of further studies to evaluate the precise role of IL-32 in IBD and CD.

### IL-32 in chronic obstructive pulmonary disease

Chronic obstructive pulmonary disease (COPD) is currently one of the leading causes of morbidity and mortality globally. COPD is a progressive disease occurring as an inflammatory response to toxic particles or gases [56], and careful estimates predict that it will be the third major cause of death by the year 2020. COPD lessens the quality of life, causes frequent hospital admissions, and ultimately enhances the risk of death [57-59]. In the US alone, the annual costs of COPD are approximately $50 billion, and most of these costs are due to exacerbations requiring hospitalization. The etiology of COPD points toward the interactions between genetic factors and various environmental factors, predominantly cigarette smoking [60]. More than 90% of cases of COPD currently are due to chronic cigarette smoking in westernized countries, which sets off an inflammatory response in peripheral lung tissues as well as in the larger bronchi. But a number of recent studies have reported a significant prevalence of COPD among non-smokers and those who have never smoked. Various studies have reported the persistence of chronic inflammation throughout the airways, pulmonary vasculature, lung parenchyma, and even outside the lungs [61,62]. A variety of factors, including the environmental indoor pollution because of use of biomass fuel consumption and coal, may be important causes of it [56,63,64]. COPD is characterized by constriction and obstruction of air passage ways and continual inflammation in pulmonary parenchymal tissues [57,65,66]. This inflammatory process is much pronounced and persistent even after quitting smoking in those susceptible smokers who develop COPD. The pathogenic mechanisms of this inflammation are not fully understood yet but various researchers have reported macrophages, T cells, and neutrophils as being important players [18,66-72]. The major inflammatory mediators in the progression of COPD were found to be respiratory epithelial cells. Multiple factors including smoking, infection and proteases have been reported by numerous researchers to be involved in the activation of airway epithelial cells in COPD [66,73-75]. These activated cells secrete a vast array of molecules prominently growth factors (GM-CSF and TGF-β), inflammatory cytokines (TNF-α, some members of IL-7 family and IL-12) and chemokines (CCL2, CXCL5, and CXCL10) [65,76,77]. Interferon gamma (IFN-γ*)* induces IL-32 expression in monocytes and epithelial cells, which through the activation of two key pathways, namely, NF-κB and p38 MAPK, results in the induction of an array of other proinflammatory cytokines and chemokines, including TNF-α, IL-8 and MIP-2 [5], which are involved in the disease progression in COPD patients. Calabrese *et al.* [78] were the first to report the effect of IL-32 in COPD-affected smokers. For this purpose, smokers with COPD were compared with non-COPD smokers and nonsmokers. The expression of IL-32 in lung tissue of COPD patients was found to be increased and correlated with the degree of airflow obstruction *in vivo*. IL-32/ TNF-α was suggested to play a key role in the progression of the immune response in COPD inflammation. Marked increased in IL-32 expression clearly showed role of IL-32 in the enhancement of the immune response in COPD, with a possible impact on disease progression . Kudo *et al.* [79] reported an increase in the expression of IL-32 due to oxidative stress (H_2_O_2_) and inflammation in human bronchial epithelial cells. A significant increase in IL-32 expression in lung samples and plasma of a similar cohort of COPD patients has been reported [78,80].

## Conclusions

Recently, the complex crosstalk between the immune system and IL-32 has become a topic of hot debate. From above data, it could be concluded that IL-32 is a key player, which exerts its biological functions intracellularly, inducing the expression of various pro-inflammatory cytokines and, hence, contributing to the progression and pathogenesis of various diseased conditions and systemic infections. Despite the recent progress, a lot of hidden potentials and features of this mysterious cytokine remain to be revealed. Currently, it is a challenge to understand the balance between its beneficial and pathological roles after administration or inhibition of this critical cytokine. However, the current status of research indicates a wide therapeutic potential of IL-32 in the medical area, in the near future.

## Abbreviations

FLS: fibroblast-like synoviocytes
GIT: gastrointestinal tract
HIV: human immunodeficiency virus
IBD: inflammatory bowel disease
IL: interleukin
IL-32: interleukin-32
JNK: c-Jun N-terminal kinase
LPS: lipopolysaccharide
MIP-2: macrophage inflammatory protein-2
NF-κB: nuclear factor kappa-light-chain-enhancer of activated B cells
NK4: natural killer cell transcript 4
NOD: nucleotide-binding oligomerization domain-containing protein
OA: osteoarthritis
PAMPs: pathogen-associated molecular patterns
PKCδ: protein kinase Cδ
poly I:C: polyriboinosinic polyribocytidylic acid
PRRs: pattern-recognition receptors
PR3: proteinase 3
RA: rheumatoid arthritis
SEMFs: subepithelial myofibroblasts
sRANKL: soluble receptor activator of nuclear factor κ-B ligand
Syk: spleen tyrosine kinase
TLR: toll-like receptor
UC: ulcerative colitis
